# Disturbances in the experiences of embodiment related to attachment, mentalization and self-objectification in anorexia nervosa

**DOI:** 10.1186/s40337-021-00463-z

**Published:** 2021-10-23

**Authors:** Hannah Katznelson, Sarah I. F. Daniel, Stig Poulsen, Susanne Lunn, Bernadette Buhl-Nielsen, Jan Magnus Sjögren

**Affiliations:** 1 Department of Psychology, University of Copenhagen, Copenhagen, Denmark; 2 Private Practice, Copenhagen, Denmark; 3 Psychotherapy Clinic, Department of Child and Adolescent Psychiatry, Region Zealand, Denmark; 4 Unit for Eating Disorders, Capital Region of Copenhagen, Psychiatric Centre Ballerup, Ballerup, Denmark

**Keywords:** Body image, Embodiment, Anorexia nervosa, Attachment, Mentalization, Self-objectification

## Abstract

**Background:**

Body image disturbance is central to both the understanding and treatment of anorexia nervosa (AN); however, the underlying psychological processes involved are still not well understood. One way towards a better understanding of these mechanisms may be to explore the sense of embodiment in these patients in an attempt at integrating the role of the body in our understanding of the development of self in AN. It is hypothesized that difficulties in affective experiences of embodiment is related to insecure attachment, deficits in mentalization and self-objectification.

**Methods:**

Sixteen inpatients with AN were interviewed with the Mirror Interview (MI). In the interview, the individual is asked a set of questions related to thoughts and feelings about the body while standing in front of a full-length mirror. Furthermore, all patients were assessed with the Adult Attachment Interview, which was coded for both attachment and mentalization (operationalized by the Reflective Functioning scale; RF). Self-objectification was measured with the Objectified Body Consciousness Scale (OBCS).

**Results:**

Results from a multiple regression analysis showed that Global MI scores were significantly associated with Coherence of mind as an indicator of attachment, RF and scores on the OBCS.

**Conclusions:**

The study suggests that affective experiences of embodiment in patients with AN are associated with negative attachment representations, mentalizing impairments and objectified body consciousness.

**Plain English Summary:**

Body image disturbance is a key diagnostic feature in anorexia nervosa but the underlying psychological processes are poorly understood. Recently, there has been a growing interest in how disturbances in the more psychological experience of the body (embodiment) in anorexia nervosa is related to both attachment, how individuals make sense of both themselves and others and the degree to which they tend to experience ourselves from the outside. In this pilot study, this was assessed with an innovative interview, the Mirror Interview, where the individual is asked a set of question while standing in front of a full length mirror. Results showed that difficulties in embodied experiences in patients with anorexia nervosa were related to more fundamental representations of self and self-objectification. This has potential implications for both the understanding and treatment of anorexia nervosa, as disturbances in body image may be seen as an underlying factor in the development of an eating disorder.

## Background

Freud famously stated that the ego is first and foremost a bodily ego [1]. In more recent conceptualizations of the self, the role of the body has been pushed into the background in favor of more symbolic, reflective and narrative aspects related to the understanding of the self [2]. However, recently researchers have started to take a greater interest in the role of the body in terms of how embodied processes are related to both the origins and maintenance of a sense of self [2, 3]. Both body image and embodiment are multidimensional constructs that refer to the body. While body image often represents a more cognitive attention to the appearance or image of the body, embodiment refers to the more complex experience of the body as engaged in the world, or the lived body [4–7]. Thus, a sense of embodiment is not about the body in itself, but related to personal, psychological and cultural experiences as understood from the standpoint of bodily being-in-the-world [7–9].

In anorexia nervosa (AN), disturbance in body image, expressed as a dysfunction in the way in which one’s body weight or shape is experienced, is a core diagnostic criterion [10]. Empirical research has consistently shown that not only does body image disturbance motivate dietary restrictions leading to severe weight loss and associated malnutrition, but it also plays a central part in both the initiation, persistence and relapse of AN (eg., [11, 12]). Accordingly, body image disturbance is central to both the understanding and treatment of AN. Furthermore, AN is associated with impaired emotional and cognitive functioning, which may also to an extent impact body image disturbance (e.g., [13, 14]). Nonetheless, the psychological processes underlying this disturbance in body image are still poorly understood [15–17].

One way towards a better understanding of these underlying psychological processes may be to explore aspects of the sense of embodiment empirically in an attempt at integrating the role of the body in our understanding of the development of self in AN. From a philosophical point of view, the phenomenological tradition has offered great and rich insights into embodiment and its relation to the self (e.g., [18]). From a psychological position, a great deal of research has focused more on the observable aspects of body image in AN, while research on the psychological, affective experience of the body and how this relates to a person’s fundamental representations of self is still limited. This scarcity is related both to the inherent complexity of trying to capture what it is like to have a body—a feeling which is both fundamental but at the same time elusive and hard to express [19], and also related to a lack of experimental measures focused on trying to access the more deep-seated dimensions of embodied processes, which are not necessarily accessible to awareness or reliably measured by self-report [4, 20]. One approach to assessing the affective experience of embodiment is through an innovative assessment, the Mirror Interview (MI) [21], which appears to be a promising clinical and research measure focusing both on how the body is represented and expressed. This paper will explore the affective experience of embodiment in patients with AN, and to see whether this experience is related both to different representations of self and aspects of objectified body consciousness, thereby contributing to the understanding of body image disturbance in this particular group of patients.

### Attachment, mentalization and the role of the body

One important aspect of the experience of the body has to do with how we come to think and feel about our bodies—a process is influenced by multiple factors such as biology, socio-cultural aspects, peers and parental influence, and include a wide range of perceptual, affective and cognitive components [22]. More recently, this has been investigated through the lens of parental attachment and the related concept of mentalization [23]. Attachment theory, developed by Bowlby [24], offers a theoretical and empirical model to explain the relationship between early experiences and adult personality functioning. According to the theory, children develop certain expectations about both themselves and others based on their experiences with caregivers. These lay the foundation for the development of *internal working models* (IWM), which are complex mental representations of how the child experiences him or herself along with an expectation about how others should treat him or her. Dependent on the quality of the caregiving environment, children consequently develop either secure or insecure patterns of attachment. Secure attachment is developed through a relationship with responsive and dependable parental figures and is characterized by positive perceptions of both oneself and others. In insecure attachment, on the other hand, the individual has developed more negative perceptions of the self, while experiencing others as either rejecting or unloving. Hence, these different patterns of attachment (or IWMs) have a great influence on the way we feel about ourselves, our representation of self and our sense of self-esteem throughout the lifespan [24–26].

Following this, it seems plausible that early caregiving experiences also have an impact on the way we feel about our bodies, i.e. that the type of care, touch, gaze, and holding a child receives will influence the way the child’s subjective body is experienced. In that sense, it can be argued that “*attachment needs are, first and foremost, body-based needs*” [27]: p. 4. It is thus proposed that core organizing assumptions of the self, developed from these early interactions with attachment figures, may be woven/entwined into the bodily representations formed by the child, thus influencing a global sense of body esteem [27]. Furthermore, individuals with more positive IWMs will have more stable feelings of general self-worth making them less likely to seek confirmation externally, as this is experienced from within the self [28]. This would in turn create a resilience in the face of adverse experiences, including the experience of failing to live up to cultural ideals of beauty such as the thin-ideal, which dominate the Western world.

Empirically, only few studies have examined this possible indirect relationship between attachment and body representations. Cheng and Mallinkrodt [29] found that lack of parental emotional caring bonds was associated with anxious adult attachment in women, and furthermore that this was associated with a tendency to internalize culturally prescribed beauty ideals leading to increased dissatisfaction with one’s body image. Similarly, Cash et al. [30] examined general adult attachment and romantic attachment in relation to body image and found that secure attachment was positively related to body satisfaction and furthermore that with regards to romantic attachment, greater anxiety was related to lower body satisfaction. In a study of adolescent girls, Patton et al. [31] also found that secure attachment to parents was associated with body satisfaction, and that perceived parental care was indirectly associated with less media internalization through more positive peer relationships. Other studies have examined the relationship between attachment classifications and AN and found that insecure attachment is far more common in patients with AN than in healthy controls [32–36], suggesting that insecure attachment can serve as a risk factor in relation to developing negative body representations in the context of eating disorder psychopathology.

Like attachment, mentalization, defined as the capacity to understand and imagine one’s own and other people’s thoughts and feelings, can be hypothesized as playing a role in how we come to think about and experience our body [23]. Mentalizing, operationalized as reflective functioning (RF), is concerned with the importance of the caregiver’s understanding of and reflection on the internal world of the small child [37], and is thus intrinsically related to the quality of attachment experiences. In other words, an individual’s understanding of both themselves and others is dependent on the degree to which their own mental states were adequately understood by caring, attentive, non-threatening adults, i.e., to the process of how “*we are first imagined to be by others”* [38]: p. 123. For the young child, being mentalized enables the integration of early developmental modes of psychic functioning into a more reflective mode where thoughts and feelings are experienced as representations and with clear boundaries between the internal and external world. According to mentalization theory, prior to this integration children are characterized by experiencing their internal world either in psychic equivalence, pretend or teleological mode [23]. In psychic equivalence mode, the internal reality is equated with the external world, whereas in the pretend mode, the child experiences an internal reality completely decoupled from the outside world. In the teleological stance, the child’s apprehension of the world is based on the physical and concrete outcomes of behavior. Children whose needs are not met are at risk of continuing to experience reality through these more primitive modes of functioning.

From a mentalization perspective, eating disorders have been conceptualized as self-disorders characterized by an underlying distortion of mental processes and capacities [23]. In a poorly integrated psyche, the role of the body in trying to create a coherent and continuous sense of self is accentuated, and affective states are thus likely to be experienced through the realm of the physical world [23]. In AN, where psychic equivalence dominates psychic funtioning, the internal world is characterized by a concreteness of thought, where there is no distinction between the inside and outside, and where mental states are represented in the physical domains of the concrete body [7]. Empirically, patients with AN have also been found to exhibit difficulties in maintaining an understanding of mental states in both themselves and others [23, 33, 36, 39, 40], thus supporting the relevance of mentalization in understanding the unsymbolized experience of the body in AN. Following this line of research, it would seem relevant to further explore empirically how mentalization relates to the affective experience of embodiment in this group of patients.

### Objectified body consciousness

Another process that has received attention as a potential vulnerability factor in relation to acquiring a healthy body image is objectified body consciousness. Objectification theory, developed by Fredrickson and Roberts [41], offers a feminist theoretical framework, which posits that women are socialized to self-objectify by imagining themselves from the perspectives of others through an internalization of cultural beauty standards. In other words, through being objectified, individuals develop the other’s perspective as their primary view of their bodily self. Such an objectification of the self can lead to an experience of a discrepancy between a person’s perceived appearance and cultural standards of beauty, which again will give rise to an experience of body shame [42]. It has been argued that high levels of objectification lead not only to a dislike of one’s own body but also to an experience of failing morally if one is not capable of shaping one’s body to fit certain beauty ideals [43]. Self-objectification can occur either at the state or trait level with trait level self-objectification referring to the relatively stable degree to which the individual has internalized an outside perspective of her body [44]. Research has also found that high levels of self-objectification are associated with body shame, restrictive eating, depressive symptoms, decrease in levels of cognitive functioning and decreased awareness of internal physical and emotional states [45–48].

In relation to eating disorders, self-objectification has been shown to contribute to the development or maintenance of the drive for thinness, internalized through media ideals, in women with eating disorders [49]. Furthermore, thin-ideal internalization and self-objectification have been found to be equally predictive in the development of body dissatisfaction and eating disorder symptoms [50]. Accordingly, it is reasonable to assume that this tendency to experience yourself from the outside, characterized by the monitoring of the body’s outward experience, would be associated with how a person experiences the subjective representation of their body, and that this tendency would be particularly pertinent for those with AN whose main motive for commencing restriction is body-image related.

### Experiencing the body in the mirror

One way to explore the relationship between attachment, mentalization and self-objectification and the individual’s sense of affective experiences of embodiment is through the Mirror Interview (MI) [21]. The MI was originally developed by Paulina Kernberg through her work with children and further developed by Bernadette Buhl-Nielsen, Lina Normandin and Miriam Steele for use in a clinical and research context with adolescents and adults as well. In the interview, the individual is asked a set of questions related to thoughts and feelings about the body while standing in front of a full-length mirror. The questions are intended to tap into experiences of being in one’s body, indications of body representations and personality structure, including aspects of identity, self and object representations and affects [3, 21]. In its original form, the MI was inspired by Winnicott’s [51] theory of the mother’s function as a mirror of the infant’s emotional experiences as crucial to the development of a sense of self. The premise of the interview is thus based on the idea that looking at oneself in an actual mirror, which symbolically represents the mothering function, elicits feelings related to the experience of being seen in early attachment relationships. While Kernberg conceived of the mirror’s mirroring function in terms of an attachment perspective, it also has implications from an objectification theoretical point of view as well, since looking at a mirror also elicits an element of seeing yourself from the outside. While looking in the mirror, the interviewee must thus manage both the active process of seeing with the passive experience of being seen [21]. The interview thus provides the individual with the experience of taking in the visual image of the physical body while having to reflect upon the affective and intersubjective experience of living-as-a-body. This process, it is postulated, taps into the affective aspects of the individual’s sense of embodiment, or the feeling of what it is like to have a body, as this is expressed both verbally and non-verbally. The coding system is designed to assess both the verbal and non-verbal expressions of this experience and makes it possible to obtain an objective assessment of an individual’s affective reaction regarding their experience of their embodied selves [20].

Thus far, research applying the MI is still in its infancy. A set of pilot studies using the MI have found associations between RF, mother’s attachment representations and their toddler daughters’ body representations [52], between maternal attachment representations and their middle childhood daughters’ body representations [53], and between parent representations on the MI and amount of disordered eating [54]. The MI has also been used to assess the impact of sexual abuse on children’s embodied self-experience [20]. Furthermore, the MI has been found to be able to significantly distinguish between healthy adolescents and those with personality disorder [55, 56]. These different findings suggest that the MI taps into important aspects of functioning related to underlying representations of body and self.

### The present study

Based on the theoretical and empirical material presented above, the aim of the current study was to conduct a pilot study focusing on affective experiences of embodiment, as assessed with the MI, in patients with AN, and to investigate how this would be related to more standardized measures of attachment, mentalization and self-objectification in this specific population which is characterized by intense problems in terms of body image dissatisfaction. The hypothesis was that negative embodiment (Global MI scores) would be associated with low levels of coherence on the Adult Attachment Interview (as a measure of attachment insecurity), lower levels of mentalization and high degree of self-objectification. More descriptively, the study also aimed to examine the subscales on the MI and eating disorder symptoms as well, but due to the small sample of the study, we had no specific hypothesis with regard to these aspects.

## Methods

### Participants

All participants (N = 16) were in-patients, recruited at the unit for eating disorders, Psychiatric Center X, X, with an ICD-11 diagnosis of anorexia nervosa (10 patients with typical AN and 6 with atypical AN). The mean age was 29.6 years (age range: 18–48, *SD* = 10.5) and six of the patients were above 30 years of age, whereas the remainder were between 18 and 30 years. The mean body mass index at the time of assessment was 17.6 kg (*SD* = 2.2) and the median duration of illness was 12.6 years (*SD* = 10.8). Ten patients had previously received in-patient treatment for AN. Exclusion criteria were suspected autism spectrum disorder, an emerging disorder within the schizophrenic spectrum, a BMI (kg/m^2^) less than 15 (to minimize the effect of extreme starvation and malnutrition), and difficulties in speaking and understanding language X.

### Procedure

All participants were recruited from the unit over an 8-month period by the first author. All patients at the clinic were given both oral and written information about the study, and those who agreed to participate all gave their prior informed consent to participate. Interview measures were conducted by the first author and symptomatology measures and BMI were assessed by the hospital staff. All assessments took place at the hospital. The study was approved by the Regional Ethics Committee of the Capital Region of X, the Ethics Committee at the Department of Psychology, University of X (record number H-15012537, addendum 71,647), and was part of the PROspective Longitudinal all-comer inclusion study in Eating Disorders (PROLED).

### Measures

*Adult Attachment Interview* (AAI) [57]. The AAI is a semistructured interview that focuses on the subject’s state of mind with regard to early attachment relationships. It consists of 20 questions with an emphasis on the relationship to primary caregivers in childhood and the effect these relationships might have had on the adult personality. The interviews were administered by a trained psychologist and subsequently recorded and transcribed verbatim. Interviews were rated by reliable and trained coders. Inter-rater agreement on four-way comparisons on six cases (37.5%) was good with Cohen’s kappa = 0.714 (*p* < 0.012).

*Reflective Function Scale* (RF) [58]. The AAI interviews were independently scored using the RF coding system. The RF scale ranges from − 1 to 9 and measures an individual’s capacity to mentalize in the context of attachment relationships. *Negative RF* (− 1) indicates an anti-reflective or bizarre type of RF, whereas *lacking RF* (1) refers to absent or distorting/self-serving RF. *Questionable or low RF* (3) is characterized by naïve-simplistic, over-analytical or miscellaneous RF. *Ordinary RF* (5) is assigned to interviews representing a coherent, if relatively simple, model of mind, whereas *Marked RF* (7) indicates a stable psychological model of mind in relation to both oneself and others. *Exceptional RF* (9) refers to interviews characterized by complex and sophisticated mentalization processes. AAI transcripts were rated for RF by trained and reliable coders. Interrater reliability on 6 cases (37.5%) was good with an Intra-Class Correlation (ICC) = 0.85.

*Mirror Interview* (MI) [21]. The Mirror Interview is a structured interview developed to tap into the affective experience of embodiment and body representations. The interview is conducted with the subject standing in front of a full-length mirror, while being asked a set of questions. The questions are related to how individuals feel about themselves, positive and negative attributions of the body, representations of parental figures with regard to the body, and perspectives on self-integration and alienation. The interview takes approximately 20 min, and is video-recorded. The rating system consists of 26 codes, which are grouped into six categories—Non-verbal, Affects, Self-worth, Relatedness, Cognition and Self-integration. Coding is done using a scale from 1 to 5 with specific scoring criteria for each code, as specified in the coding manual, but throughout the coding system 1 represents very negative responses, whereas 5 represents more positive, normative responses. A Global MI score was obtained by adding the scores of the six categories. All interviews were rated by reliable and trained coders. Interrater agreement on 6 cases (37.5%) was high with an ICC of 0.903 (*p* < 0.000).

*The Objectified Body Consciousness Scale* (OBCS) [42]. Developed to measure trait-level self-objectification, the OBCS is a questionnaire with 24 items grouped into three subscales—Body Shame (e.g., “When I can’t control my weight, I feel like something must be wrong with me.”), Control Beliefs (e.g., “I think a person can look pretty much how they want to if they are willing to work at it.”), and Body Surveillance (e.g., “During the day, I think about how I look many times.”). Participants respond on a 7-point Likert scale ranging from “strongly agree” to “strongly disagree”. Originally, the OBCS was validated on undergraduate women with reported coefficient alphas for the subscales of 0.89 (Surveillance), 0.75 (Body Shame) and 0.72 (Control Beliefs) [42].

*Eating Disorder Examination Questionnaire* (EDE-Q4) [59]. The EDE-Q4 consists of 36 items measuring attitudes and behavior towards eating disorder problems in the last 28 days. The measure generates a Global scale and four subscales (Restraint, Eating Concern, Weight Concern, and Shape Concern). Items comprising the Global scale and subscales are rated on a 0–6 scale with higher scores reflecting greater severity of symptoms. The EDE-Q has shown good inter-rater reliability, internal consistency, and discriminant validity [59].

## Results

### Statistical analysis

Data were analyzed using a multiple regression analysis to test whether attachment, mentalization and self-objectification were significantly related to Global MI scores. Furthermore, Pearson correlation analyses was used to examine the relationship between the study variables. Analyses were conducted in SPSS, version 25.0.

### Distribution of attachment patterns and RF

Basic characteristics of the sample are shown in Table 1 and the distribution of attachment classifications is shown in Table 2. Of the 16 patients, 9 (56.3%) were rated dismissing (Ds), 4 (25%) were rated preoccupied (E), and 3 (18.8%) were rated as secure (F) in terms of attachment classification. Three (18.8%) were rated unresolved (U)/cannot classify (CC). Mean RF scores were 3.19 (*SD* = 1.45).

**Table 1:** Sample characteristics

Variable	N = 16	
	N	%
Currently in a relationship	4	25
Children	4	25
Years of education		
9 or less (ISCED level 2)	2	12.5
10–12 (ISCED level 3)	9	56.25
14 (ISCED level 4)	2	12.5
15 (ISCED level 5–6)	2	12.5
17 or more (ISCED level 7–8)	1	6.25

	M	SD
Age	29.6	10.5
BMI	17.6	2.2
Duration of illness	12.6	10.8
EDE-Q	3.66	1.45
RF	3.19	1.22

ISCED = International standard classification of education by UNESCO

**Table 2:** Distribution of attachment patterns

	Three category		Four category		Five category	
Classification	Frequency	%	Frequency	%	Frequency	%
Secure	3	18.8	3	18.8	3	18.8
Dismissing	9	56.3	8	50	8	50
Preoccupied	4	25	2	12.5	2	12.5
Unresolved			3	18.8	2	12.5
Cannot Classify					1	6.3
Total	1	100	16	100	16	100

### Regression analysis of mirror interview

The continuous Coherence of mind scale from the AAI coding system was used as a measure of attachment security. A stepwise multiple linear regression was calculated to investigate the relationship between Global MI scores and coherence of mind, RF and OBCS. A significant regression analysis was found (F (3, 11) = 15.671, *p* < 0.001) with a *R*^2^ of 0.810 for Global MI scores. Both coherence of mind (*p* < 0.007), RF (*p* < 0.001) and OBCS (*p* < 0.001) were significantly associated with Global MI.

Pearson’s correlations between the different variables are shown in Table 3. There was a strong significant correlation between coherence of mind and RF (0.629, *p* < 0.01), suggesting that the coherence of mind scale of the AAI and the Reflective Function Scale measures were related, but not identical, constructs. Furthermore, Global MI scores were also correlated with EDE-Q scores (0.619, *p* < 0.01). On a subscale level of the MI, RF was highly correlated with Affects (0.704, *p* < 0.01) and Self-worth (0.551, *p* < 0.05), and scores on the OBCS was highly correlated with Self-worth (− 0.653, *p* < 0.01), Cognition (− 0.520, *p* < 0.05) and Self-integration (− 0.608, *p* < 0.05).

**Table 3:** Summary of Pearson correlations, means, and standard deviations for scores on coherence of mind, RF, OBCS, EDE-Q, global MI and MI subscales

Measure	1	2	3	4	5	6	7	8	9	10	11	M	SD
1. Coherence of Mind	–											3.72	1.46
2. RF	.629**	–										3.19	1.22
3. OBCS	.280	.197	–									122.07	23.51
4. EDE-Q	.224	.408	.748**	–								3.66	1.45
5. Global score MI	− .098	− .554*	− .676**	− .619*	–							15.86	1.93
6. Non-verbal	− .102	− .351	− .358	− .051	.640**	–						2.64	0.33
7. Affects	− .328	.704**	− .427	− .462	.836**	.505*	–					2.77	0.54
8. Self-worth	− .354	− .551*	− .653**	− .693**	.772**	.222	.703**	–				2.34	0.44
9. Relatedness	.097	− .457	− .407	− .614*	− .784**	.461	.517*	.498*	–			2.70	0.43
10. Cognition	.251	− .104	− .520*	− .176	.670**	.607*	.459	.311	.394	–		2.83	0.41
11. Self-integration	.066	− .213	− .608*	− .686**	.719**	.203	.434	.585*	.615*	.294	–	2.58	0.45

RF = Reflective functioning scale; OBCS = objectified body consciousness scale; EDE-Q = eating disorder examination questionnaire; MI = mirror interview. *p < 0.05, **p < 0.01

## Discussion

This pilot study set out to examine the role of attachment, mentalization and self-objectification in relation to experiences of embodiment in a sample of female inpatients with AN. The sample was characterized by a high level of severity in symptoms and a long history of AN. In accordance with previous studies [32, 33, 36], we found that the majority of patients were classified as insecure with regard to attachment representation, and that most of these were classified as dismissing. In total, 13 individuals (81.3%) were classified as insecure with nine (56.3%) of these being dismissing and four (25%) preoccupied. Only three patients were rated as secure (18.8%). Furthermore, three patients were classified as unresolved (18.8%). Previous research has differed in relation to the incidence of unresolved traumatic experiences in AN. In a study by Ward et al. [36], 50% (n = 10) of the patients were classified as unresolved with respect to loss or trauma, whereas Barone and Guiducci’s [32] did not find any in their sample of patients with AN (n = 10).

We further found that mentalizing was impaired in the sample with a mean RF of 3.19, suggesting a less developed ability to differentiate between physical and emotional states and to differentiate and understand mental states in both themselves and others. This supports previous research, which have found comparable low levels of RF in samples with patients with AN [33, 36, 40].

With regards to our main hypothesis that lower global MI scores would be associated with lower levels of coherence of mind, lower levels of mentalization and a higher degree of self-objectification, results showed that Global MI scores were significantly associated with coherence of mind (as an indicator of attachment security), RF and scores on the OBCS. This supports the general assumption that affective experiences of embodiment in patients with AN are associated with adverse early caregiving experiences, both in terms of negative attachment representations and impaired mentalizing capacities, and with the degree to which these patients self-objectify in terms of their experience of themselves. More descriptively, we also found that Global MI scores were correlated with eating disorder symptomatology (assessed with the EDE-Q), which suggest that difficulties in the sense of embodiment potentially play a role in eating disorder psychopathology. On a subscale level of the MI, the subscales Affects, Self-worth (which also covers body esteem), Cognition and Self-integration were particularly highly correlated with RF and OBCS, potentially indicating that these areas of functioning may be of specific interest in relation to AN.

Taken together, results suggest that difficulties related to embodiment are potentially important in the understanding of AN, as research has shown that body image disturbances in AN are difficult to alter and may be a persistent trait-like factor [60–62]. The results of the present study indicate that this tenacity of body image disturbances may to some extent be a result of fundamental disturbances in the development of experiences of embodiment related to both attachment patterns, mentalizing deficits and objectified body awareness.

Furthermore, the results may also be discussed in the context of the concept of embodied mentalization, as put forward by Fotopoulou and Tsakiris [2]. Fotopoulou and Tsakiris [2] argue that the formation of the self is reliant on the social mentalization of the body and its homeostatic needs. In that sense, social interactions between caregiver and child do not only shape that reflective self and the associated notions of affect regulatory and social cognitive capacities. Instead, they forward the idea that “*the most minimal aspects of selfhood*, namely the feeling of being an embodied, agentive subject, *are fundamentally shaped by embodied interactions with other people* in early infancy and beyond” [2]: p. 6. This idea underlines the importance of incorporating the body into the study of the development of self, both clinically and in terms of research. Following this line of thought, the MI can be proposed as an objective assessment tool, which tries to bridge this gap, in its attempt to capture the affective embodied experience evoked by taking in one’s visual image in the mirror. From a clinical perspective, it is also important to note that for some patients suffering from AN being exposed to their image in a mirror can be experienced as challenging, which probably caused some patients to abstain from participating in the study in the first place. For all the patients who participated in the study, the procedure was talked through before the start of the interview, and after the interview any emotional distress potentially arisen from the exposure was talked through in depth either with the interviewer or with hospital staff in order to make sure that the experience was made clinically meaningful for the participant in relation to their specific situation, illness and treatment plan.

A strength of the study is related to its use of gold-standard assessments of both attachment and RF, as measured with the AAI, along with the validated measure of the OBCS. However, while the results from the present study suggest that the MI can be reliably used as a coding system with valid psychometric properties and good interrater reliability, additional research is warranted to further validate the psychometric properties of the interview, for example examining test–retest reliability. Thus, the findings should be interpreted with caution and considered preliminary due to the limitation of the present study design. The first obvious limitation is related to the small sample size. Even though samples with patients with AN in an attachment context are often quite small (e.g., [32, 36]), the study would need replication in a larger sample. The specific sample also limits the generalizability of the study very substantially and further studies with larger sample sizes are clearly needed. Furthermore, correlation does not entail causality, and consequently the relationship between the variables still remains hypothetical. Moreover, there is no control group in the present study, and future related research involving control groups would be of great interest, particular in relation to the effect on patients who have recovered from AN.

## Conclusion

In summary, the findings of the study seem to suggest that it is possible to identity difficulties in the affective experience of embodiment in patients with AN by using the MI. Through the use of the mirror, the MI activates affective responses and elicits both intense and spontaneous behaviors and reactions, which are otherwise difficult to access and assess within an objective, empirical research framework. The study showed that these difficulties in the experience of embodiment were associated with standardized measures of attachment, mentalization and self-objectification, thus supporting the overall hypothesis that difficulties in embodied experiences in patients with AN are related to more fundamental representations of self and an objectified body consciousness in this particular sample. However, future research involving replication in a larger sample might also be able to further contribute to a better understanding of the nuances of the complex interaction between these different factors in relation to the development of self.

By implication, this could also be of potential importance in the development of our understanding of body image disturbances in relation to both the etiology and maintenance of AN, as research have shown this core symptom to be a pervasive characteristic of AN and may even be an underlying factor in the development of an eating disorder [60, 62]. Thus, the study also has potential clinical implication for the treatment of AN in terms of identifying these particular difficulties in the affective experiences of embodiment in individual patients and in terms of guiding specific therapeutic interventions which explicitly address these disturbances.

## Data Availability

The datasets used and/or analysed during the current study are available from the corresponding author on reasonable request.

## Abbreviations

AN: Anorexia nervosa
IWM: Internal working models
RF: Reflective functioning
AAI: Adult attachment interview
MI: Mirror interview
OBCS: Objectified body consciousness scale
